# An Attack on the Army Medical Service

**Published:** 1917-04-21

**Authors:** 


### AN ATTACK ON THE ARMY MEDICAL SERVICE.

Two recent issues of the *World* have contained very uncompromising attacks upon the conduct and management of the Army Medical Service, written apparently by two doctors who have inside acquaintance with the working of it. The matter is one of some public importance; and since it is unlikely that the A.M.S. authorities will take any public notice of anonymous attacks, it is perhaps all the more necessary for independent witnesses to testify, as far as in them lies, whether the attacks have or have not a strong basis of justification.

To start with, neither of the two attacks in question appears on the surface to be marshalled with much ability. The charges brought are vague, some of the criticisms are of a carping nature, and there are few suggestions for constructive reform. It is, of course, possible that these deficiencies are partly due to reluctance in the minds of the two critics to give any clue to their own identity, a reluctance which is by no means necessarily a sign of bad faith or of personal delinquency. We fancy that a skilled controversialist, of the *John Bull* pattern, could make out a much better case against the A.M.S. than the two correspondents of the *World* have done.

It is, on the whole, true, as suggested by one of these two critics, that the Army Medical Service has all through the campaign enjoyed a very "good press." Besides, it must be conceded that the Service has been highly efficient, not merely by comparison with wars of the past, but actually. The Service has deserved, on the whole, the "good press" which it has unquestionably enjoyed. The *World's* doctor argues that this success has been achieved in spite of, not because of, the excellence of the Army Medical Service; that it has been the work of the civilian doctor, not that of his official Army superior, that has been the main factor in the success of the treatment of the sick and wounded.

On the whole it is probable that there is truth in the *World's* contention. Unquestionably the civilian medical men in their thousands have borne the burden and heat of the day, whereas the regular R.A.M.C. and A.M.S. have enjoyed so far the lion's share of the credit and the rewards. But the Medical Service is not the only branch of the Army in which the same complaint is heard; and it is one of the ways of the world that its prizes are reserved rather for those who set others to work than to the most competent and hardest workers themselves. To a great extent it is inevitable that the administrative posts in our vastly expanded Army shall be in the hands of those whose previous training had, nominally at all events, qualified them best to hold them. It is perfectly true that the best brains of the profession did not invariably gravitate into the R.A.M.C.; and thus it comes about that some nonentities and nincompoops occupy positions of command over many men of brilliant brains and attainments. This fact is mainly the result of our British state of total unpreparedness for war, a fault due to the whole nation, and especially to its politicians, rather than to any one profession or to any one section of the old Army.

Another charge brought is that there is too much "eyewash" in the Army Medical Service. Certainly there is—far too much of it; and the reason is that too many of the most senior officers—the Surgeon-Generals and A.D.s M.S.—are impressed by eyewash and unable to tell it from genuine efficiency. That this is the case few men who have held commissions in the R.A.M.C. will be disposed to deny; but at the same time it must be added that there is probably no more "eyewash" in the Army Medical Service than in other branches of the Army—or for that matter in the Civil Service or other Government Departments. Although it is certainly widespread, there are good men amongst the Regular R.A.M.C., who with the civilian doctors in the Corps are much less infected with it, and carry out their work with all possible energy and skill for its own sake and for that of the wounded.

Yet another criticism that is voiced is the old one of wasted resources: of putting highly trained specialists to trivial tasks, of keeping large numbers of officers in idleness abroad while home hospitals are starved of staff, and of similar maladministration. Here, again, there is substance in the complaint, but how much of substance it is difficult to say. This complaint has been voiced many times, from the first year of the war onwards; but it does not seem that any great improvement or any marked economy of resources has been brought about. Altogether, in our judgment, the authorities will do well to heed these critics, even though their presentation of their case is imperfect. After all, the efficency of the Medical Services should stand above all personal or regimental considerations; and the military medical authorities should be willing to learn from anyone that is capable of pointing out improvements or of indicating defects.

